# A Longer Interstimulus Interval Yields Better Learning in Adults and Young Adolescents

**DOI:** 10.3389/fnbeh.2018.00299

**Published:** 2018-12-03

**Authors:** Katarina Kjell, Karolina Löwgren, Anders Rasmussen

**Affiliations:** ^1^Department of Psychology, Lund University, Lund, Sweden; ^2^Logopedics, Phoniatrics and Audiology, Department of Clinical Sciences, Lund University, Lund, Sweden; ^3^The Linnaeus Centre Thinking in Time: Cognition, Communication and Learning, Lund University, Lund, Sweden; ^4^Associative Learning, Department of Experimental Medical Science, Faculty of Medicine, Lund University, Lund, Sweden; ^5^Erasmus Medical Center, Department of Neuroscience, Rotterdam, Netherlands

**Keywords:** eyeblink conditioning, interstimulus interval (ISI), adolescent, comparative analaysis, classical conditioning, cerebellum, timing, motor learning

## Abstract

Eyeblink conditioning is one of the most popular experimental paradigms for studying the neural mechanisms underlying learning and memory. A key parameter in eyeblink conditioning is the interstimulus interval (ISI), the time between the onset of the conditional stimulus (CS) and the onset of the unconditional stimulus (US). Though previous studies have examined how the ISI affects learning there is no clear consensus concerning which ISI is most effective and different researchers use different ISIs. Importantly, the brain undergoes changes throughout life with significant cerebellar growth in adolescents, which could mean that different ISIs might be called for in children, adolescents and adults. Moreover, the fact that animals are often trained with a shorter ISI than humans make direct comparisons problematic. In this study, we compared eyeblink conditioning in young adolescents aged 10–15 and adults using one short ISI (300 ms) and one long ISI (500 ms). The results demonstrate that young adolescents and adults produce a higher percentage of CRs when they are trained with a 500 ms ISI compared to a 300 ms ISI. The results also show that learning is better in the adults, especially for the shorter ISI.

## Introduction

### Eyeblink Conditioning

Eyeblink conditioning is a simple form of learning in which a subject is presented with a neutral conditional stimulus (CS), such as a tone or a light, followed by a blink-eliciting unconditional stimulus (US) such as an air puff or an electrical shock delivered to the periorbital region. Repeatedly pairing the CS and the US eventually causes the CS to trigger a conditional blink response (CR), even in the absence of the US.

Several lines of converging evidence from animals show that the cerebellum is necessary for eyeblink conditioning. Animals lacking their entire telencephalon can still acquire conditioned blink responses (Norman et al., 1974), while removing the cerebellum and leaving the rest of the brain intact eliminates CRs (McCormick and Thompson, 1984). Neurophysiological data from mice (Heiney et al., 2014; ten Brinke et al., 2015), rabbits (Halverson et al., 2015) and ferrets (Rasmussen et al., 2015; Jirenhed and Hesslow, 2016), further emphasizes the cerebellums vital role in eyeblink conditioning.

Patients with cerebellar ataxias and cerebellar lesions exhibit deficits in eyeblink conditioning. This suggests that the cerebellum is essential for eyeblink conditioning in humans as well (Daum et al., 1993; Gerwig et al., 2005; Timmann et al., 2005). Nevertheless, evidence shows that eyeblink conditioning also activates other brain regions, not only the cerebellum. For example, PET scans show activity in the cerebral cortex (Blaxton et al., 1996), and fMRI scans also show activation of the hippocampus during eyeblink conditioning even if that effect is stronger when using a trace conditioning paradigm (Cheng et al., 2008). Based on this evidence Steinmetz (2000) argues that while eyeblink conditioning will not occur without the cerebellum, the learning normally occurs in concert with other brain regions.

### The Interstimulus Interval

An important feature of the CR is its adaptive timing. Participants do no blink immediately after the tone. Rather, they blink near the onset of the US (Kehoe and Macrae, 2002). The time between the CS onset and the US onset is known as the interstimulus interval (ISI): a key parameter in eyeblink conditioning. In addition to determining the timing of CRs (Steinmetz et al., 2011), studies show that the ISI also affects the learning rate (Kehoe and Macrae, 2002). No learning occurs if the ISI is shorter than 100 ms (Kimble, 1947; McAllister, 1953a; Salafia et al., 1980; Wetmore et al., 2014). The performance also declines if the ISI is more than 1 s (McAllister, 1953a,b; Schneiderman and Gormezano, 1964).

An unexplored pattern in the literature is that the optimal ISI for training animals such as rabbits, rats, mice and ferrets, seems to be around 300 ms (Schneiderman and Gormezano, 1964; Gormezano et al., 1983; Kehoe and Macrae, 2002), whereas humans appear to learn more efficiently when the ISI is longer than 300 ms (Kimble, 1947; McAllister, 1953b; Frings et al., 2010). Moreover, an ISI that would be considered normal or long in the animal literature is considered short when the subjects are human. For example, one study deemed a 440 ms ISI “short” and a 880 ms ISI “long” (Gerwig et al., 2005). Other studies on humans have used ISIs exceeding 1 s (Herbert et al., 2003; Weidemann et al., 2016), which is almost never used in the animal literature. In general, it seems that researchers studying eyeblink conditioning in humans consistently use longer ISIs than researchers studying animals. The lack of consensus regarding the most effective ISI in humans and the fact that different research groups use different ISIs reduces the generalizability of findings and potentially hinders scientific progress.

### Cerebellar Development and Eyeblink Conditioning in Children and Adolescents

The cerebellum undergoes changes throughout life, with substantial development occurring in childhood and adolescents. During this period, high-order cerebellar dependent cognitive functions develop and mature in co-occurrence with the increasing volume of the cerebellum (Tiemeier et al., 2010). These anatomical changes correspond to findings in the eyeblink conditioning literature as younger children (aged 4–12) typically perform worse on conditioning than adults (Reeb-Sutherland and Fox, 2015), whilst older children (aged 9–12) has a learning rate more similar to adults (Löwgren et al., 2017).

These findings have led researchers to propose eyeblink conditioning as a non-invasive biomarker for cerebellar abnormalities in neurodevelopmental disorders in both children and adults (Reeb-Sutherland and Fox, 2015). However, to determine if deficits in timing is a potential biomarker for neurodevelopmental disorders, further understanding of normal cerebellar functioning is called for. To the best of our knowledge, no previous study has systematically examined the appropriate ISI in typically developed adolescents. This study examines age-related differences in learning between adolescents and adults using the two different ISIs, 300 ms and 500 ms. These two ISIs were chosen because a 300 ms ISI approximates the ISI used in most animal studies while ~500 ms is normal in studies on humans.

## Materials and Methods

### Ethics and Participants

All participants and legal guardians were informed about and gave their written consent to the experimental protocol. As a token of our gratitude, the participants received cinema ticket coupons following their participation. The regional ethics committee in Lund, Sweden, approved this study and all associated procedures (H15 2009/383). Our initial samples consisted of 106 adolescents recruited from a local elementary school and 45 adults recruited from the student population at Lund University and via acquaintances. Of the adolescents, 10 were excluded from the analysis because they had received or were being assessed for a neuropsychiatric disorder, or because they wanted to stop the testing (usually because they found the air-puff unpleasant). Following data collection, we detected that there was a significant difference in the average age of the adolescents trained with 300 ms and those trained with 500 ms. To ensure that this age difference did not confound our results and to homogenize the age range of the adolescents trained with different ISIs we also excluded data from the youngest and the oldest adolescents in the sample. Thus, the final analysis included data from 61 adolescents and 45 adults. Descriptive statistics from the two populations included in the final analysis are provided in Table 1. For more information see Supplementary Material Datasheet 1.

**Table 1:** Descriptive statistics.

	Adolescents (*n* = 61)	Adults (*n* = 45)
Age (mean ± SD)	12.2 ± 0.8	28.2 ± 8.5
Sex	Females = 28; Males = 33	Females = 28; Males = 17
ISI	300 ms = 33; 500 ms = 28	300 ms = 19; 500 ms = 26

### Materials

**Figure 1:**
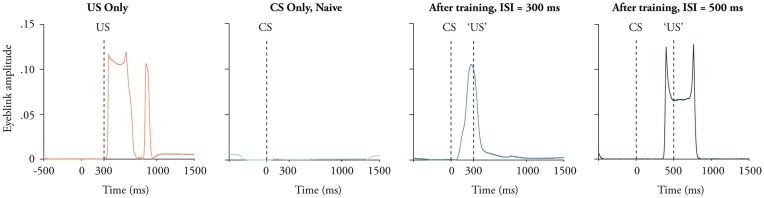
Examples of eye-movements in response to the conditional stimulus (CS) and the unconditional stimulus (US). Presenting only the US elicits a short latency blink reflex (red). Before training the CS elicits no response (green), but after approximately 100 trials the same stimulus elicits a conditional blink response whose peak approximately matches the interstimulus interval (ISI). The exact shape of the traces depends on the position of the magnet and the GMR sensor. This explains why the trace to the right has two peaks even though the trace next to it does not.

To record eyelid movements, we attached a small round neodymium magnet (diameter: 3 mm; thickness: 1 mm height) on the right eyelid of the participant using a small piece of double cohesive tape. We recorded movements of the magnet, and the eyelid (see Figure 1), by placing a sensitive GMR chip near the eye of the participant. Magnetic field measurements from the GMR were sampled at 1,000 Hz and sent to the computer via a Mirco1401 (Cambridge Electronics Design), or a national instruments AD converter. The same AD converter was used to trigger tones, delivered via speakers or headphones, and air-puffs which were generated by a compressor or by connecting a pressurized N2 gas tube to digitally controlled pressure valves D132202 solenoid valve (AirCom). The amplitude of the tone was set so that the participants could hear the tone clearly but did not blink. The pressure of the air-puff was set so that it elicited a clear blink response without causing the participant any discomfort. The pressure was usually between 0.5 and 1 bar. For distraction and entertainment, all participants watched a cartoon or a movie during the testing. For further details, we refer the reader to our previous publications. For further details about the training of the adults we refer the reader to Löwgren et al. (2017). For further details about the procedure that the adolescents went through we refer the reader to Rasmussen and Jirenhed (2017).

### Procedure

The adolescents were tested in a room at their school and the adults were tested in the humanities lab at Lund University. After placing the magnet and calibrating the stimuli we instructed the participants to focus on the movie and try not to control their eyelids after which we started the movie and the training. The adults received a total of 80 trials (8 blocks of 10 trials) and the adolescents received 100 trials (10 blocks of 10 trials). Adolescents received ~20% randomly interspersed probe trials in which the CS but not the US was presented. The adults received 25% probes on pre-determined trials, mainly towards the end of training (Löwgren et al., 2017). The ISI was 300 ms or 500 ms, and the intertrial interval was 15 ± 5 s.

### Analysis

Data from the adolescents were stored in Spike2 files. These files, containing waveform data sampled at 1,000 Hz and stimulus triggers, were exported to Matlab (Mathworks). Custom made algorithms were used to identify the onset and the peak of blink responses on each trial. A response was categorized as a CR if it had an onset at least 100 ms after the CS and before the onset of the US. These two criteria were used on CS alone (probe) trials as well as on paired trials. After this automatic analysis, one experimenter visually inspected the eyelid movements on each trial. Clear categorization errors were corrected manually. Data from the adults were sampled and analyzed using custom LabView software. The learning rate (percent CRs) was based on both paired and CS alone trials whereas the topographical CR analysis was based exclusively on CS alone trials to avoid interference from the US. For statistical analysis of CR acquisition, we used a combination of *t*-tests on individual training blocks and mixed effects models to analyze the entire learning curve while also taking into account the fact that different individuals have different means. Statistical analysis of CR timing we used *t*-tests on all valid CRs from the entire training period. Unless stated otherwise averages are expressed as Mean ± SD.

## Results

### A 500 ms ISI Yields Better Learning

To examine the effect of training, age, and the ISI on learning, we modeled the CR percentage using a mixed effects model with the ISI, block and age category (adult vs. adolescent) as fixed effects and the participant as a random effect. Linear mixed effects models have several advantages compared to repeated measures analysis of variances (ANOVAs). The procedure is statistically robust; it takes into account the differences between individuals; and it allows you to include participants with missing data points (Krueger and Tian, 2004).

The mixed model showed that training led to an increase in the CR percentage. For each subsequent block the CR percentage increased by 1.8 ± 0.2%, *p* < 0.0001, 95% CI [1.4%, 2.2%]. The mixed effects model also showed that lengthening the ISI from 300 ms to 500 ms, resulted in a statistically significant increase in the CR percentage of 25 ± 4.5%, *p* < 0.0001, 95% CI [16%, 34%]. Finally, the model showed that adults produced more CRs than the adolescents: 19 ± 4.6%, *p* < 0.0001, 95% CI [9.5%, 28%].

**Figure 2:**
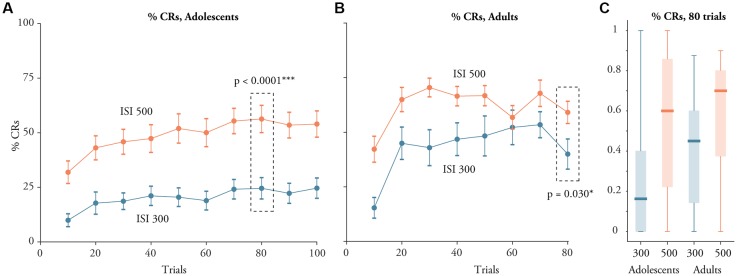
Learning in young adolescents aged 10–15 and adults with a 300 ms or 500 ms ISI. **(A)** Percent CRs (Mean ± SEM) on 10 successive blocks each consisting of 10 trials among the adolescents. **(B)** Percent CRs on eight successive blocks each consisting of 10 trials among adults. **(C)** Boxplots illustrating the distribution of the percentage of CRs in block eight for adolescents and adults. The bottom line represents the minimum value, the lower edge of the box is the first quartile, the line represents the median, the top of the box is the third quartile and the top line is the maximum value.

The difference between adults and adolescents was however primarily due to the poor learning in adolescents trained with a 300 ms ISI. We compared two simplified mixed models, one with the ISI and block as fixed effects and one which also included the interaction between the ISI and the block. The model that also included the interaction was a significantly better model, *p* = 0.0040. This shows that the shorter ISI (300 ms) was associated with a greater dip in learning among the adolescents than among the adults. This pattern can be seen in Figure 2 where there is a larger difference between adolescents and adults trained with a 300 ms ISI than there is between adolescents and adults trained with a 500 ms ISI.

### Post-training Comparisons

For post-training analysis we compared the CR percentage in block 8 (trials 71–80). In block 8, the adolescents and the adults had received the same number of trials and approximately the same number of CS alone trials. Hence the percentage of CRs should be comparable across all groups.

In agreement with the linear mixed effect model, adolescents and adults trained with a 500 ms ISI produced more CRs than those trained with a 300 ms ISI (Figure 2A). In block 8, adolescents trained with a 300 ms ISI produced CRs on 25 ± 28% of the trials, while adolescents trained with a 500 ms ISI produced CRs on 61 ± 30% of the trials. An independent *t*-test confirmed that adolescents trained with a 500 ms ISI produced more CRs on block 8 than adolescents trained with a 300 ms ISI: *t*_(56)_ = 4.59, *p* < 0.0001, *d* = 1.2. The same pattern was evident among the adults (Figure 2B). Adults trained with a 300 ms ISI produced CRs on 40 ± 30 percent of the trials whereas those trained with a 500 ms ISI produced CRs on 59 ± 26% of the trials in block 8: *t*_(42)_ = 2.25, *p* = 0.0296, *d* = 0.68.

### Learning Occurred Mainly in the First Block

The learning curves in Figure 2 suggest that much of the learning occurred early. To examine this statistically we did *t*-tests comparing the percentage of CRs on block 8 with the percentage of CRs on successive blocks from the start of training. For adults and adolescents and for both ISIs there was a significant difference between the percentage of CRs on block 8 and block 1 (*p* < 0.036). In contrast, there was no difference between block 8 and block 2 (*p* > 0.3), except for adolescents trained with a 500 ms ISI (*p* = 0.043). This shows that participants generally reached a learning asymptote within the first 10–20 trials, irrespective of age and the ISI (see Supplementary Material Datasheet 2 for more information).

Similarly, the difference in the CR percentage for the two different ISIs was evident even in the first block of training (see Figure 2). This difference was statistically significant for the adolescents, *t*_(59)_ = 3.83, *p* = 0.0003, *d* = 0.967, as well as for the adults, *t*_(59)_ = 3.33, *p* = 0.0018, *d* = 1.03. In contrast, there were no significant differences between adolescents and adults in the percentage of CRs produced in block 8, after 80 trials. This was statistically verified by two independent *t*-tests, one for training with a 300 ms ISI: *t*_(48)_ = 1.723, *p* = 0.091; and one for training with a 500 ms ISI: *t*_(50)_ = 0.19, *p* = 0.85. Yet, the absence of an effect in block 8 may partly be due to the fact that the adults received more CS-alone trials on this block, resulting in the dip in CR percentage visible in Figure 2B.

### Timing of Conditioned Responses

**Figure 3:**
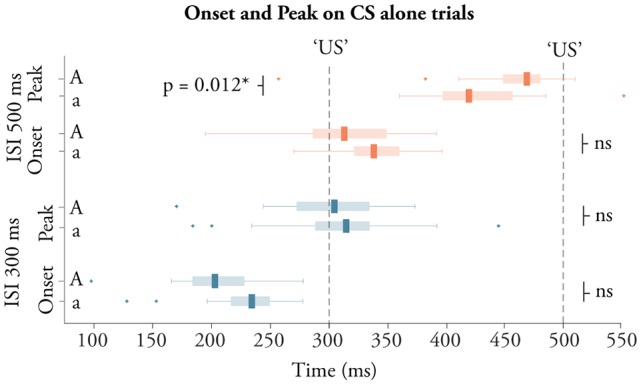
CR timing. Boxplots illustrating the timing of the onset and peak of conditioned responses elicited during training with a 500 ms ISI (red boxes) and 300 ms (blue boxes), for adolescents (bottom box in each pair) and adults (top box in each pair).

Conditioned responses are adaptively timed; their onset and peak depend on the ISI used. This was also the case here. As illustrated in Figure 3, young adolescents and adults produced CRs with a shorter latency to the onset and peak of the CR during training with a 300 ms ISI compared to training with a 500 ms ISI (all *p* < 0.0001, all *d* > 2.0). Adolescents trained with a 300 ms ISI produced CRs with an average onset latency of 219 ± 51 ms and a peak latency of 313 ± 50 ms. For adults, the onset was 204 ± 40 ms and the peak was 299 ± 47 ms. Young adolescents trained with a 500 ms ISI produced CRs with an onset of 337 ± 32 ms and a peak latency of 424 ± 40 ms. For adults trained with a 500 ms ISI, the onset was 317 ± 44 ms and the peak latency was 456 ± 49 ms. Comparing the temporal profile of the CRs between adolescents and adults revealed one significant difference in the peak latency following training with the longer 500 ms ISI, *t*_(52)_ = 2.59, *p* = 0.013, *d* = 0.70.

## Discussion

### A Longer ISI Yields Better Learning

The main finding of this study is that adults and young adolescents acquire conditioned blink responses faster and reach a higher percentage of CRs following training with a 500 ms ISI compared to a 300 ms ISI. Although a 500 ms ISI was associated with better learning in both groups, adults on average learned faster than the adolescents, especially with the shorter, 300 ms, ISI. As expected, training with a 500 ms ISI resulted in CRs with a longer latency to onset and peak compared to CRs following training with a 300 ms ISI. One weakness of this study is that adults received four more CS alone trials than the adolescents on average. We cannot exclude that this difference affected the learning. However, the protocol dissimilarities are unlikely to explain the differences in learning because the adults—who learned faster—actually received fewer paired trials (25% vs. 20%).

Our finding that a 500 ms ISI yields better learning is consistent with the bulk of previous studies. Kimble (1947) reported that within the range of 100–400 ms, a longer ISI yielded better learning. Similarly, Ebel and Prokasy (1963), reported higher levels of CRs following training with a 500 and 800 ms ISI compared to a 200 ms ISI. In contrast to our results McAllister (1953b), found that a 250 ms ISI yielded slightly better conditioning than a 450 ms ISI, while an even longer ISI (700–1,200 ms) resulted in lower rates of CRs. Steinmetz et al. (2011) found slightly better conditioning with a 350 ms ISI compared to an 850 ms ISI.

Thus, with the exception of McAllister (1953b), the literature and our results suggests that the optimum ISI for humans lies somewhere between 350 ms and 800 ms. Consequently, if researchers want their human participants to reach a high level of CRs quickly, it is advisable to use an ISI that is ~350 ms or higher, as is the norm (Thurling et al., 2015; Allen et al., 2018).

### Adults vs. Young Adolescents

Comparing the adolescents with the adults reveals that adults produced more CRs than the adolescents. In our previous study, we found that among children there was a positive correlation between the percentage of CRs and the age of the children. However, children above the age of nine performed on par with the adults (Löwgren et al., 2017). At first glance, our finding here that the adults performed better than the young adolescents seems to be at odds with our prior results. However, from Figure 2 it is clear that adolescents trained with a 300 ms ISI drag down the average for the adolescents. In contrast, the performance of the adolescents trained with a 500 ms ISI—the interval we used in our previous study—looks almost similar to that of the adults. This ISI-age interaction was verified statistically by comparing two mixed models, one which included the interaction and one which did not. The fact that there is a greater difference between adults and young adolescents when using a short ISI, indicates that the neural mechanisms may be somewhat distinct (see below). The fact that cerebellar development continues throughout adolescence (Tiemeier et al., 2010), could mean that the minimum ISI to which participants are able to learn depends on age.

### Contrasts With Studies on Animals

Compared to studies on humans, studies on animals have usually employed a shorter ISI, in the region of 200–500 ms (Halverson et al., 2015; ten Brinke et al., 2015; Zucca et al., 2016; Giovannucci et al., 2017; Rasmussen et al., 2018). Mice which are becoming increasingly popular as an experimental animal for studying eyeblink conditioning have, to our knowledge, never been trained with an ISI exceeding 500 ms. Therefore, we do not know if mice can learn when the ISI is longer than 500 ms, even though ISIs of that duration are commonly used when training humans. These normative differences in the duration of the ISI between studies on animals and humans make direct comparisons between humans and other species problematic. The simple fact that all studies that have investigated the role of the ISI suggest that it plays at least some role, implies that the mechanisms are not exactly the same and hence we should be open to the possibility that a 300 ms and a 500 ms ISI relies on overlapping but somewhat distinct mechanisms. Our observation that young adolescents, as well as adults, perform poorly with a 300 ms ISI highlights this concern further and suggests that more research is necessary before we can seamlessly generalize between different species.

### Inconsistent Learning Patterns

A difference between humans and animals when it comes to eyeblink conditioning is that humans display greater individual differences in their performance. The four boxplots in Figure 2C reveals that the percentage of CRs in the last session ranges from zero to more than 90%. In other words, there were adolescents and adults, who after 80 trials produced CRs on almost every trial; but there were also individuals who did not produce any CRs at all, even after 80 trials. This was the case for both ISIs. Moreover, the learning curves (Figures 2A,B) show that some individuals produce >30% CRs in the first block of 10 trials. A similarly high rate of CRs shortly after training initiation is evident in many other studies examining eyeblink conditioning in humans. The exact rate varies from ~20% to 30% (Frings et al., 2010; Steinmetz and Rice, 2010; Tran et al., 2017), all the way to >60% CRs in the first 10 trials (Solomon et al., 1989). Equally strange is the fact that some humans do not seem to learn at all. As mentioned, some individuals produced no CRs at all. It seems unlikely that poor performers have cerebellar deficits since such deficits would probably lead to severe clinical symptoms.

Individual differences, mediated by factors such as locomotion (Albergaria et al., 2018), are also present in other species. However, compared to humans, other species appear to learn at a more predictable rate. Other species generally do not produce CRs in the first block; and other species generally do not reach 100% CRs after 80 trials.

### Influence of Volition

How can we explain the erratic learning pattern among humans? We believe that a plausible explanation is to be found in the experimental design. Prior studies have shown that the instructions given to the participants influence the learning pattern (Gormezano and Moore, 1962; Nicholls and Kimble, 1964). Moreover, if prompted to do so, participants can produce voluntary blink responses that are indistinguishable from classically conditioned blink responses (Rasmussen and Jirenhed, 2017). If the generation of a CR is, to some extent, under voluntary control then this can explain both high rates of CRs in the beginning of training as well as the absence of CRs. Whether the participants would do so voluntarily in the sense that they are aware of it is another matter, and we would still need to explain why the degree of voluntary meddling differs between different groups.

## Author Contributions

AR, KK and KL conceived and designed the experiments, and performed the experiments. AR and KL analyzed the data. AR made the figures with suggestions from KK and KL and wrote the manuscript with input from KK and KL.

## Conflict of Interest Statement

The authors declare that the research was conducted in the absence of any commercial or financial relationships that could be construed as a potential conflict of interest.
