# Supplementary material for: A Longer Interstimulus Interval Yields Better Learning in Adults and Young Adolescents
Source: Front Behav Neurosci. 2018 Dec 3;12:299. doi: 10.3389/fnbeh.2018.00299 (PMC6286956; doi:10.3389/fnbeh.2018.00299)
Supplement: Supplementary file 2 [file Data_Sheet_2.docx]

Supplementary material

# Linear mixed effects model (output from matlab)

Linear mixed-effects model fit by ML

Model information:

Number of observations 958

Fixed effects coefficients 4

Random effects coefficients 106

Covariance parameters 2

Formula:

CRs ~ 1 + Block + Group2 + ISI2 + (1 | FP)

Model fit statistics:

AIC BIC LogLikelihood Deviance

-335.52 -306.33 173.76 -347.52

Fixed effects coefficients (95% CIs):

Name Estimate SE tStat DF pValue Lower Upper

'(Intercept)' 0.12646 0.038153 3.3146 954 0.00095243 0.051588 0.20134

'Block' 0.018163 0.002084 8.7153 954 1.2668e-17 0.014073 0.022253

'Group2' 0.1859 0.046156 4.0276 954 6.083e-05 0.095317 0.27647

'ISI2' 0.25068 0.045568 5.5013 954 4.8439e-08 0.16126 0.34011

Random effects covariance parameters (95% CIs):

Group: FP (106 Levels)

Name1 Name2 Type Estimate Lower Upper

'(Intercept)' '(Intercept)' 'std' 0.22526 0.19512 0.26006

Group: Error

Name Estimate Lower Upper

'Res Std' 0.17312 0.1651 0.18154

The same pattern was evident when we analyzed the entire acquisition phase using simple linear regression with CR percentage as the dependent variable and the ISI and the block as the two independent variables. A significant regression equation was found for the adolescents (F(2,596) = 99.874, p < 0.00001, R^2^ = 0.251), as well as for the adults (F(2,356) = 21.197, p < 0.00001, R^2^ = 0.106).

# Descriptive statistics

Adults (yrs) = 28.2704 +/- 8.5095

Adolescents (yrs) = 12.1626 +/- 0.82277

Adult Males: 17 Females 28

Adolescents Males: 33 Females 28

Adults ISI 300 = 19, 500: 26

Adolescents ISI 300 = 33, 500: 28

# T-tests on age

300 vs 500, Adolescents Age

Mean Age ISI 300: 11.9798 +/- 0.83021

Mean Age ISI 500: 12.378 +/- 0.77363

Independent ttest, t(59) = -1.9256, p = 0.058984, d = 0.49622

# T-tests on %CR, ISI 300ms vs ISI 500ms

## All subjects, ISI 300 vs ISI 500, %CRs

Mean CR percentage block 8 All Subjects ISI 300: 0.3059 +/- 0.29488

Mean CR percentage block 8 All Subjects 500: 0.59839 +/- 0.2781

Independent ttest, t(100) = -5.1553, p = 1.2802e-06, d = 1.0205

## Adolescents, ISI 300 vs ISI 500, %CRs

Mean CR percentage block 8 Adolescents ISI 300: 0.25306 +/- 0.28292

Mean CR percentage block 8 Adolescents ISI 500: 0.606 +/- 0.30072

Independent ttest, t(56) = -4.5935, p = 2.5175e-05, d = 1.2088

## Adults, ISI 300 vs ISI 500, %CRs

Mean CR percentage block 8 Adults ISI 300: 0.39985 +/- 0.3001

Mean CR percentage block 8 Adults ISI 500: 0.59078 +/- 0.25926

Independent ttest, t(42) = -2.2519, p = 0.029613, d = 0.68088

# T-tests on %CR, adults vs adolescents

## ISI 300, adults vs adolescents, %CRs

Mean CR percentage block 8 Adults ISI 300: 0.39985 +/- 0.3001

Mean CR percentage block 8 Adolescents ISI 300: 0.25306 +/- 0.28292

Independent ttest, t(48) = 1.7231, p = 0.091303, d = 0.5033

## ISI 500, adults vs adolescents, %CRs

Mean CR percentage block 8 Adults ISI 500: 0.59078 +/- 0.25926

Mean CR percentage block 8 Adolescents ISI 500: 0.606 +/- 0.30072

Independent ttest, t(50) = -0.19542, p = 0.84586, d = 0.054198

# T-tests on the CR onset

## ISI 300 vs 500, adolescents, CR onset

Mean Onset Adolescents ISI 300: 719.9554 +/- 50.9825

Mean Onset Adolescents ISI 500: 836.8926 +/- 31.5506

Independent ttest, t(50) = -10.0975, p = 1.1587e-13, d = 2.7583

## ISI 300 vs 500, adults, CR onset

Mean Onset Adult ISI 300: 703.5706 +/- 40.2915

Mean Onset Adult ISI 500: 817.6008 +/- 44.4949

Independent ttest, t(42) = -8.6803, p = 6.4249e-11, d = 2.6865

## ISI 300, adults vs adolescents, CR onset

Mean Onset Adult ISI 300: 703.5706 +/- 40.2915

Mean Onset Adolescents ISI 300: 719.9554 +/- 50.9825

Independent ttest, t(40) = -1.1243, p = 0.26759, d = 0.35659

## ISI 500, adults vs adolescents, CR onset

Mean Onset Adult ISI 500: 817.6008 +/- 44.4949

Mean Onset Adolescents ISI 500: 836.8926 +/- 31.5506

Independent ttest, t(52) = -1.8483, p = 0.070246, d = 0.50018

# T-tests on the CR peak

## ISI 300 vs 500, adolescents, CR peak

Mean Peak Adolescents ISI 300: 813.7471 +/- 50.0749

Mean Peak Adolescents ISI 500: 924.8557 +/- 40.2272

Independent ttest, t(50) = -8.871, p = 7.7119e-12, d = 2.4463

## ISI 300 vs 500, adults, CR peak

Mean Peak Adult ISI 300: 798.6843 +/- 47.1295

Mean Peak Adult ISI 500: 956.1298 +/- 48.5065

Independent ttest, t(42) = -10.7079, p = 1.4001e-13, d = 3.2923

## ISI 300, adults vs adolescents, CR peak

Mean Peak Adult ISI 300: 798.6843 +/- 47.1295

Mean Peak Adolescents ISI 300: 813.7471 +/- 50.0749

Independent ttest, t(40) = -0.98902, p = 0.3286, d = 0.30978

## ISI 500, adults vs adolescents, CR peak

Mean Peak Adult ISI 500: 956.1298 +/- 48.5065

Mean Peak Adolescents ISI 500: 924.8557 +/- 40.2272

Independent ttest, t(52) = 2.5862, p = 0.012543, d = 0.70185

# Learning asymptote

## Adults, ISI 300

Block 1 vs Block 8: t(35) = 2.9169, p = 0.0061349**, d = 0.95455

Block 2 vs Block 8: t(35) = -0.48142, p = 0.63322, d = 0.15852, - not significant

Block 3 vs Block 8: t(35) = -0.26904, p = 0.78948, d = 0.088714 - not significant

## Adults, ISI 500

Block 1 vs Block 8: t(50) = 2.1562, p = 0.035906*, d = 0.59801

Block 2 vs Block 8: t(50) = -0.7825, p = 0.43761, d = 0.21703 - not significant

Block 3 vs Block 8: t(50) = -1.7106, p = 0.093348, d = 0.47444088714 - not significant

## Adolescents, ISI 300

Block 1 vs Block 8: t(62) = 2.5791, p = 0.012292*, d = 0.64477

Block 2 vs Block 8: t(62) = 0.96479, p = 0.3384, d = 0.2412 - not significant

Block 3 vs Block 8: t(62) = 0.96542, p = 0.33809, d = 0.24135 - not significant

## Adolescents, ISI 500

Block 1 vs Block 8: t(50) = 3.3833, p = 0.0013995*, d = 0.93835, d = 0.64477

Block 2 vs Block 8: t(50) = 2.0772, p = 0.042938*, d = 0.57612 - not significant

Block 3 vs Block 8: t(50) = 1.5204, p = 0.13471, d = 0.42168 - not significant
